# Counting Oceanians of Non-European, Non-Asian Descent (ONENA) in the South Pacific to Make Them Count in Global Health

**DOI:** 10.3390/tropicalmed4030114

**Published:** 2019-08-09

**Authors:** Arnaud Tarantola, Paul F. Horwood, Cyrille Goarant, Bertrand Buffière, Solène Bertrand, Onofre Edwin A. Merilles, Thierry Pedron, Elise Klement-Frutos, Philippe Sansonetti, Lluis Quintana-Murci, Vincent Richard

**Affiliations:** 1 Epidemiology Unit, Institut Pasteur de Nouvelle-Calédonie, BP 61–98845 Nouméa cedex, New Caledonia; 2 Virology and Viral Diseases, College of Public Health, Medical and Veterinary Sciences, Division of Tropical Health and Medicine, James Cook University, Townsville, QLD 4811, Australia; 3 Leptospirosis Research and Expertise Unit, Institut Pasteur de Nouvelle-Calédonie, BP 61–98845 Nouméa cedex, New Caledonia; 4 Communauté du Pacifique/Pacific Community, 95 Promenade Roger Laroque, BP D5, 98848 Nouméa, New Caledonia; 5 Unité de Pathogénie Microbienne Moléculaire, INSERM U1202, Institut Pasteur, 28 rue du Docteur Roux, CEDEX 15, 75724 Paris, France; 6 Internal Medicine and Infectious Diseases Department, Centre Hospitalier Territorial, 110 Boulevard Joseph Wamytan, Dumbéa Sur Mer 98835, Nouvelle-Calédonie; 7 Chaire de Microbiologie et Maladies Infectieuses, Collège de France, 11 square Marcelin Berthelot, 75005 Paris, France; 8 Human Evolutionary Genetics Unit, Institut Pasteur, 25–28 Rue du Docteur Roux, 75015 Paris, France; 9 Institut Pasteur de Nouvelle-Calédonie, BP 61—98845 Nouméa cedex, New Caledonia

**Keywords:** medical genetics, diabetes mellitus, type 2, non-communicable diseases, global health, ethnic groups, indigenous health, infectious diseases, nutritional and metabolic diseases, epidemiology, pacific islands

## Abstract

Several diseases and vulnerabilities associated with genetic or microbial factors are more frequent among populations of Oceanian, Non-European, Non-Asian descent (ONENA). ONENA are specific and have long been isolated geographically. To our knowledge, there are no published official, quantitative, aggregated data on the populations impacted by these excess vulnerabilities in Oceania. We searched official census reports for updated estimates of the total population for each of the Pacific Island Countries and Territories (including Australia) and the US State of Hawaii, privileging local official statistical or censual sources. We multiplied the most recent total population estimate by the cumulative percentage of the ONENA population as determined in official reports. Including Australia and the US State of Hawaii, Oceania counts 27 countries and territories, populated in 2016 by approximately 41 M inhabitants (17 M not counting Australia) among which approximately 12.5 M (11.6 M not counting Australia) consider themselves of entire or partial ONENA ancestry. Specific genetic and microbiome traits of ONENA may be unique and need further investigation to adjust risk estimates, risk prevention, diagnostic and therapeutic strategies, to the benefit of populations in the Pacific and beyond.

## 1. Introduction

Some gene polymorphisms are found more frequently in certain human groups compared to other populations [1,2]. This is particularly true among ethnic groups or populations that have lived long in isolation for geographic, cultural or other historical reasons [3,4]. In most instances, these polymorphisms are simply normal-functioning variants of genes [5]. Although the influence of lifestyle and dietary habits seems dominant, ethnic origin and corresponding gene polymorphisms may also influence the microbiome [6,7,8]. Microbiome studies have evidenced associations with inflammation and certain diseases or vulnerabilities [9,10]. 

For the purpose of this paper, we define Oceania as all countries, commonwealths and territories located in the South Pacific, including Australia, New Zealand and the U.S. State of Hawaii. Archaeological and genetic data suggest that the Oceania region was settled in two main historical waves of migration: 1) the Australo-Papuan (Indigenous Australian and Melanesian) people settled in the region possibly more than 60,000 years ago, shortly after the first ‘out-of-Africa’ migration; 2) Austronesian (Polynesian and Micronesian) people settled in the region less than 4000 years ago, probably originating from East Asia [11]. Indigenous Australians (Aboriginal and Torres Strait Islander people of Australia) and other Pacific islanders (Melanesians, Micronesians and Polynesians) seem to constitute very specific human populations both in terms of genetic traits [11,12] and microbiome [13]. Some of these genetic traits may have selected to confer a comparative advantage during perilous transoceanic migrations or in an often-challenging environment.

Several diseases known to be associated with genetic risk factors, however, are more frequent among populations of Oceanian, Non-European, Non-Asian (ONENA) ancestry [14,15,16,17]. In various territories in the region (Australia, New Zealand and Hawaii), ONENA are disproportionally affected by infectious, as well as non-communicable diseases, when compared to their non-ONENA compatriots [18,19]. Although social and cultural factors are major contributors, these vulnerabilities may be exacerbated in some instances by genetic or microbial risk factors. This disproportionate health burden is well documented by public health studies [20].

Surprisingly, we could find no official aggregated quantitative data on the number of people which may be impacted by these vulnerabilities in Oceania. As a preliminary step to further research we, therefore, sought to estimate the 2016 total population of ONENA in Oceania (including Hawaiians and Aboriginal and Torres Strait Islander Australians) to whom these estimated excess health risks may apply.

## 2. Materials and Methods 

We listed all countries, commonwealths and territories in Oceania: The Pacific Islands, Australia and Torres Straits Islands, and the US State of Hawaii. We then searched official census reports available for updated estimates of the total population of each of these countries or territories, privileging local official statistical or censual sources. When local estimates were dated prior to 2016 then the United Nations population projections for 2016 [21] were used. We also searched these reports to find the most recent official estimates of population distribution by ethnic group for each Oceanian country or territory. In order not to underestimate potential vulnerabilities, the percentage of populations of self-declared mixed ancestry including ONENA were added to that of persons entirely of self-declared ONENA ancestry. We then multiplied the 2016 total population estimate by the cumulative percentage of ONENA population to obtain an absolute number. Although correct and reliable, the resulting current ONENA population estimates were rounded off to the nearest hundred so as not to give a misleading idea of precision. Data were entered, percentages computed and added and multiplied by population totals using a spreadsheet (Microsoft Excel^®^, Redmond, WA, USA) and results were mapped using ArcGIS 10.6.1 (ESRI. Redlands, CA, USA). 

## 3. Results

**Figure 1:**
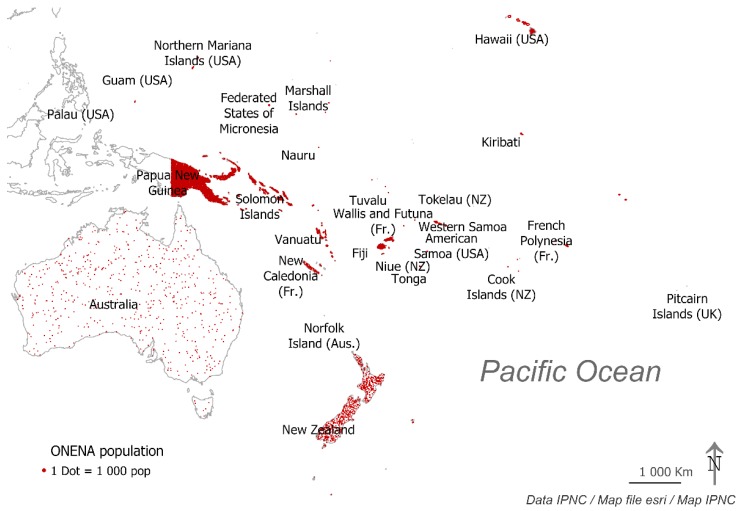
Oceania and relative estimates of population subgroups considered/self-declared as descending entirely or in part of Oceanian of Non-European, Non-Asian descent (ONENA) populations, Pacific region and US State of Hawaii, 2016. Points represent 1 000 pop and are randomly distributed, not georeferenced.

Including Australia and the US State of Hawaii, Oceania counts 27 countries and territories, populated by approximately 41 M inhabitants including 24 M in Australia and 17 in other countries and territories. Among these, approximately 12.5 M (11.6 M not counting Australia) consider themselves or are considered entirely or partly of ONENA ancestry (Table 1 and Figure 1). 

These populations are very diverse: ONENA were reported as being entirely or partly descending from more than 30 different population subgroups. When these subgroups are considered as a whole, the cumulated population of ONENA is in the range of countries such as Rwanda, Tunisia, Cuba or Belgium and would be situated in the 68th percentile of populations of countries or inhabited areas listed in a 2015 United Nations report [22]. Most ONENA live in Papua New Guinea, New Zealand or Australia, but—with the notable exception of Papua New Guinea—the territories in which ONENA people constitute the majority of the population are small. Depending on the territory considered, ONENA accounted for 3% to 100% of the territory’s population. Most territories were home to several distinct ONENA groups. In some territories, populations were categorized as “Pacific Islanders”. In some others such as New Caledonia, censuses proposed an “undetermined” or otherwise culturally/politically important but ethnically imprecise category (“New Caledonian”).

## 4. Discussion

However approximate, this is to our knowledge, the first published and detailed estimate of the number of Oceanians of Non-European, Non-Asian ancestry (Micronesians, Melanesians and Polynesians and Aboriginal and Torres Strait Islander Australians) living in countries or territories composed of the predominantly South Pacific islands, New Zealand, Australia and Torres Islands and the US State of Hawaii.

Oceania’s population is youthful [77] and on the rise, overall. According to United Nations estimates, the total population in Oceania should reach approximately 47 M by 2030 and 57 M by 2050 [22]. The absolute number of the ONENA population, however, is on the rise in some territories, but decreasing in others due to migration [51] or due in part to the consequences of climate change [78]. Following European colonization, ONENA populations suffered tremendous mortality due to the introduction of infectious diseases such as influenza [19,79], dysentery or measles [80]. Successive measles outbreaks, for example, led to population collapses in some Pacific Island communities by 80%–90% within 1–2 generations following first contact with Europeans [81]. Although much of this mortality may be explained by the lack of lifetime immunity in previously unexposed island populations [17], population genetic and perhaps microbiotic homogeneity likely also contributed to the exceptionally high mortality rates due to influenza [19] and other infections. 

Today, the morbidity and mortality burden remains higher among ONENA compared to non-ONENA people in the South Pacific, whether from infectious diseases (influenza [82], leptospirosis [83] or rheumatic heart disease [84], etc.) or especially, from non-communicable diseases (diabetes, obesity [85], etc.), which in Oceania present some of the highest incidence and/or prevalence worldwide [20]. These diseases also incur high direct or indirect health costs, especially for Oceanian countries or territories already facing significant economic, demographic and climate-driven challenges [86,87].

Many of these diseases are now known to be associated with genetic risk factors [20]. The latter have been identified thanks to many studies conducted on diseases and genetic or microbiotic risk factors in industrialized countries, among populations of overwhelmingly European or Asian descent [88,89]. Despite the high cost paid to these diseases, ONENA, however, are usually not represented in these large-scale genetics or microbiome studies as they represent a small proportion of the population and study cohorts, especially in Europe or the Continental United States [90].

### 4.1. Bias and Limitations

Our study may suffer from bias and limitations. First, mixed ancestry may not reflect allele distribution—even in Mendelian inheritance—and census reports cannot provide information as to whether individuals with mixed ancestry inherited ONENA or European alleles, for example. The default allocation of individuals of mixed ancestry to ONENA may, therefore, have overestimated vulnerabilities’ allele frequency. The role of mitochondrial genomes in ONENA also remains unclear. Census reports from Oceania, however, show that persons declaring mixed ancestry are in much smaller proportions than those declaring single-ONENA ancestry (Table 1). Second, ancestry is usually self-declared by census participants, which also may have overrepresented certain population subgroups. Studies, however, have shown that self-declared ancestry is well correlated with gene markers, at least those predictive of origin at the “continental” scale [88,91]. Third, 39% of the population of Easter Island are of Andean ancestry. Although these qualify as non-European and non-Asian and likely have specific vulnerabilities, they were not included in the ONENA totals which focus on long-residing populations of Oceania. This, however, was applicable only to a small population of a few thousand. Fourth, some census participants declared themselves as “undeclared”, “not stated”, etc., or were declared as “other”. The ancestry of these participants, therefore, cannot be accurately determined. We report census data as published; therefore, these were not removed from the population denominators. As opposed to the first and second points, this may somewhat underestimate the proportion of ONENA in the entire population in some countries or territories. Finally, documented ethnicity may sometimes be purposefully misrepresented due to social/political agendas. Most Oceanian countries and territories, however, are stable democracies and in most ONENA represent the overwhelming majority, with little interest to misestimate populations of European or Asian ancestry. We, therefore, conclude that our review provides the most reliable estimate possible of the true number of ONENA.

### 4.2. Public Health Implications

We considered ethnic origin only to quantitatively estimate populations that could ultimately benefit from targeted allelic or microbiotic vulnerabilities research, potentially leading to improved, adjusted—even “precision”—prevention or therapeutic programs. Albeit debated, the consequences—in a given environment—of host genetics, of the gut microbiome, of their effects on immune function [10], on obesity [92], type 2 diabetes [93,94], metabolic, inflammatory, allergic or other diseases such as colorectal cancer are the subject of increasing research [95,96,97]. Although translating research into tangible results is always a challenge [98], a clearer picture of the number of ONENA people and their health vulnerabilities is needed to meet the health challenges they face. This can also help document human genetic diversity, ultimately contributing to improved prevention and care of infectious and non-communicable diseases [17], response to certain drugs [99] or to toxins [100] in the Pacific and beyond. Several institutions or organizations—especially in New Zealand, Australia or the US State of Hawaii—are already mandated and strive to meet this objective of adjusting the public health response to varying ethnic burdens and vulnerabilities.

## 5. Conclusions

Authorities and legal frameworks are tasked with protecting and promoting the rights of all citizens of all origins equally, without distinction, including in providing access to health and timely and adequate healthcare. Alongside other determinants of health, genetic and microbiome traits of the ONENA population need to be considered to equitably adjust risk estimates, risk prevention, diagnostic and therapeutic strategies, perhaps leading even to adjusted individual medical care [101,102], “precision medicine” or even “precision public health” initiatives [103]. Furthermore, studies which include ONENA participants in research may be important for the global community as a whole, as certain genetic polymorphisms or epigenetic profiles may be more frequent among ONENA, who may shed light, for example, on genetic factors contributing to severe influenza [17] or diabetes [91] in any human population subgroup. Although these fields of research are quickly developing, much remains to be discovered or explained. As a member of the International Network of Pasteur Institutes, the Institut Pasteur in New Caledonia will strive to contribute with other stakeholders to the improvement of health and healthcare for all in New Caledonia, in the rest of Oceania and beyond.

## Figures and Tables

**Table 1:** Estimates of total populations and of population subgroups considered/self-declared as descending entirely or in part of ONENA populations, Oceania, 2016.

Territory (Country or Commonwealth)	Most Recent Population Estimate	Year of Census or Estimate	Census or Best Estimate in 2016 *	Ethnic Groups (Adapted from Cited Sources)	Estimated** and Rounded-off 2016 ONENA Population		Ref.
					N	%	
**American Samoa (USA)**	55,519	2010	54,196	Native Pacific islander or mixed 94.4%; Asian or mixed 4.4%; White 0.9%; Other 0.1%	51,500	95%	[21,23,24]
**Australia**	24,051,000	2016	24,051,000	European/mixed European and other 91%; Asian/mixed Asian and other 6%; Indigenous Australian/mixed Indigenous Australian and Torres Straits Islanders and other 3%; Pacific Islanders 0.7%	890,000	4%	[25,26,27,28]
**Cook Islands (NZ)**	17,459	2016	17,459	Cook Island Maori 81.3%; part Cook Island Maori 6.7%; other 11.9%	15,400	88%	[29,30]
**Easter Island (Chile)**	6370	2015	6370	Rapa Nui 59.9%; Andean Chilean 39.3%; Other 0.9%	3800	60%	[31]
**Federated States of Micronesia**	102,843	2010	105,000	Chuukese or mixed 46.8%; Pohnpeian or mixed 28.3%; Kosraean or mixed 6.3%; Yapese or mixed 6.1%; Other Pacific Islanders or mixed 10.4%; Asian 1.3%; White 0.3%; Other 0.6%	102,600	98%	[21,32]
**Fiji**	869,458	2015	898,000	Chinese 0.6%; European 0.4%; iTaukei 56.8%; Part European 1.3%; Indian 37.5%; Rotuman 1.2%; Other Pacific Islanders 0.8%; Other 1.5%	529,000	59%	[21,33,34]
**French Polynesia (Fr.)**	272,800	2015	272,800	Polynesian 82.7%; Asian 4.7%%; European 11.9%	226,400	83%	[35,36]
**Guam (United States)**	162,742	2016	162,742	Chamorro (or mixed) 43.4%; other Pacific Islander (or mixed) 13.3%; Asian 34.3%; white 7.1%; other 2.1%	92,300	57%	[37,38]
**Hawaii (USA)**	1,431,603	2015	1,428,557	Asian 37.3%; Multiethnic 23.0%; White 26.7%, Native Hawaiian and Pacific Islander 9.9%; Black 2.6%; Native American 0.5%	138,800	10%	[39,40,41]
**Kiribati**	110,110	2015	114,000	I-Kiribati & mixed 98.0%; Tuvalu 0.2%; Fiji 0.1%; Australia 0.04%; New Zealand 0.6%; European 0.1%; Chinese 0.1%; Other 0.9%	108,200	98%	[42,43]
**Marshall Islands**	55,158	2011	56,400	Pacific Islanders 92.7%; Asian 3.6%; Mixed 2.7%; Other 1.2%	49,300	98%	[21,44,45,46]
**Nauru**	10,084	2011	10,000	Nauruan 90.8%; other Pacific Islander 6.1%; Asian 2.1; Other 1.0%	8400	84%	[47,48,49,50,51]
**New Caledonia (Fr.)**	268,767	2014	268,767	Kanak (Melanesian) 39.0%; European 27.1%, Wallisian & Futunian 8.2%, Tahitian 2.1%; Indonesian 1.4%; Vietnamese0.9%; Ni-Vanuatu 1%; “Caledonian”/other/undeclared 22.7%	135,000	50%	[52,53]
**New Zealand**	4,693,000	2016	4,693,000	European 66.7%; Maori 13.4%; Asian 10.6%; Pacific Islander 6.7%; other 2.6%	1,126,300	24%	[54,55]
**Niue (NZ)**	1611	2011	1600	Niuean 66.5%; Part Niuean 13.4%; Pacific Islander 8%; Asian and European 12%	1800	88%	[21,56]
**Norfolk Island (Aus.)**	1796	2011	2210	European ancestry 88%; Polynesian ancestry 12%	66	3%	[57,58,59]
**Northern Mariana Islands (USA)**	53,883	2010	55,700	Asian 49.9%; Pacific islander 34.8%; Mixed 12.7%; Other 2.5%	19,100	35%	[21,60,61]
**Palau**	17,501	2012	17,800	Palauan 72.5%; Filipino 16.3%; Chinese 1.6%; other Asian 5.0%; white 0.9%; Carolinian 1%; other Micronesian 2.4%; other 0.3%	13,000	76%	[21,62]
**Papua New Guinea**	7,620,000	2015	7,776,000	New Guinea Papuan 84%; New Guinea Melanesian 15%; other (Negrito; Polynesian; Melanesian; other) 1%	7,776,000	100%	[21,63,64,65]
**Pitcairn Islands (UK)**	57	2012	54	European; Tahitian	0	0%	[49,66]
**Samoa**	187,820	2011	194,899	Samoan or part-Samoan 98.5%; other/missing/don’t know: 1.5%	191,800	99%	[67,68]
**Solomon Islands**	642,000	2015	639,418	Melanesian 95.3%; Polynesian 3.1%; Micronesian 1.2%; other 0.3%	637,500	100%	[69]
**Tokelau (NZ)**	1499	2016	1499	Tokelauan 69.3%; Samoan 14.1%; Tuvaluan 9.2%; Other Pacific Islander 3.5%; European 2.8%; Other ethnic group or not stated 1.1%	1400	96%	[51,70,71]
**Tonga**	103,252	2011	107,000	Tongan (or mixed) 97.5%; Pacific islanders or mixed (0.7%); European 0.6%; Asian 1.0%; Other/not stated: 0.3%	105,000	98%	[21,72]
**Tuvalu**	10,782	2012	10,000	Tuvaluan or mixed Tuvaluan 99.1%; Other 0.9%	9,900	99%	[21,73]
**Vanuatu**	234,023	2009	271,000	Ni-Vanuatu 97.6%; part Ni-Vanuatu 1.1%; 1.3% other	268,500	99%	[21,74,75]
**Wallis & Futuna (Fr.)**	12,197	2013	13,000	Polynesian	13,000	100%	[21,76]
**Total**	**40,988,967**				**12,514,066**	**30%**	
**Total minus Australia**	**16,937,967**				**11,624,066**	**69%**	

* Projected 2016 estimates from the United Nations World Statistics Pocketbook 2016 [21] unless otherwise indicated; ** Rounded-off estimates obtained by multiplying the cumulative percentages of ethnic groups found in various cited sources by the total population estimated in 2014–2016 found in various sources (not shown), also cited. In practice, this sometimes means applying percentages documented in 2002 to documented or projected populations in 2015. These data, and especially their apparent precision, must be considered with caution.
